# Comment on ‘Association Between Dynapenic Obesity and Risk of Cardiovascular Disease: The Hisayama Study’ by Setoyama et al.

**DOI:** 10.1002/jcsm.13684

**Published:** 2024-12-22

**Authors:** Chang Liu, Fan Zhang, Min Cao

**Affiliations:** ^1^ Department of Emergency Longhua Hospital Shanghai University of Traditional Chinese Medicine Shanghai China; ^2^ Department of Nephrology A Longhua Hospital Shanghai University of Traditional Chinese Medicine Shanghai China

To Editor,

We have read with interest the article by Setoyama Y et al. [1] titled ‘Association of dynapenic obesity with cardiovascular disease: The Hisayama Study.’ While this study provides valuable insights into the relationship between dynapenic obesity and cardiovascular disease risk, we would like to highlight three points that warrant further discussion.

First, the authors introduce the concept of ‘dynapenic obesity’ and discuss it alongside ‘sarcopenic obesity’ in the introduction, citing multiple references related to sarcopenic obesity. However, according to the 2022 guidelines from the European Society for Clinical Nutrition and Metabolism (ESPEN) and the European Association for the Study of Obesity (EASO), sarcopenic obesity is specifically defined as the coexistence of obesity and sarcopenia [2]. In Setoyama et al.'s study, dynapenic obesity only considers handgrip strength and body mass index, whereas handgrip strength is just one dimension of assessing sarcopenia and cannot fully represent a sarcopenia diagnosis [3]. Therefore, the authors should not equate dynapenic obesity with sarcopenic obesity. This conceptual confusion may lead to misinterpretation and misapplication of the study results.

Second, this study not only spans a median follow‐up of 24 years but also assesses baseline handgrip strength and body mass index. Over such an extended follow‐up period, important variables such as participants' physical function, height, weight, dietary habits, and physical activity are likely to have changed significantly. These changes would inevitably affect the association between exposure and outcome, and we consider this crucial limitation should be emphasized more strongly, with a discussion of its potential impact on the study findings.

Third, from a statistical perspective, the study uses Cox proportional hazards models to analyse the relationship between dynapenic obesity and cardiovascular disease risk. However, given the long‐term nature of the follow‐up, the proportional hazards assumption may not hold. The impact of dynapenic obesity on cardiovascular disease risk might change over time. Therefore, we suggest that the authors consider using time‐dependent Cox models or other statistical methods suitable for long‐term follow‐up data to more accurately capture the time‐varying relationship between exposure and outcome [4].

Additionally, the study employs multiple comparisons but does not appear to have applied any correction for multiple testing. This could increase the risk of Type I errors, leading to false‐positive results. We recommend that the authors consider using appropriate methods for multiple comparison correction, such as the Bonferroni correction or false discovery rate methods [5].

In conclusion, while Setoyama et al.'s study provides important insights into the relationship between dynapenic obesity and cardiovascular disease risk, the above issues warrant further discussion and clarification to enhance the reliability and applicability of the study findings.

## Ethical Approval

The authors have nothing to report.

## Conflicts of Interest

The authors declare no conflicts of interest.

## References

[1] Y. Setoyama , T. Honda , T. Tajimi , et al., “Association Between Dynapenic Obesity and Risk of Cardiovascular Disease: The Hisayama Study,” Journal of Cachexia, Sarcopenia and Muscle (2024): Epub ahead of print. 10.1002/jcsm.13564 PMC11634510 39378156

[2] L. M. Donini , L. Busetto , S. C. Bischoff , et al., “Definition and Diagnostic Criteria for Sarcopenic Obesity: ESPEN and EASO Consensus Statement,” Clinical Nutrition 41, no. 4 (2022): 990–1000. 35227529 10.1016/j.clnu.2021.11.014

[3] A. J. Cruz‐Jentoft , G. Bahat , J. Bauer , et al., “Sarcopenia: Revised European Consensus on Definition and Diagnosis,” Age and Ageing 48, no. 1 (2019): 16–31. 30312372 10.1093/ageing/afy169 PMC6322506

[4] L. D. Fisher and D. Y. Lin , “Time‐Dependent Covariates in the Cox Proportional‐Hazards Regression Model,” Annual Review of Public Health 20 (1999): 145–157. 10.1146/annurev.publhealth.20.1.145 10352854

[5] Y. Benjamini and Y. Hochberg , “Controlling the False Discovery Rate: A Practical and Powerful Approach to Multiple Testing,” Journal of the Royal Statistical Society: Series B (Methodological) 57, no. 1 (1995): 289–300.

